# In-unit particulate matter (PM2.5) in a public housing complex and the importance of tobacco and cannabis as indoor pollutants

**DOI:** 10.1016/j.indenv.2026.100157

**Published:** 2026-02-13

**Authors:** Madeleine Wallace, Saira Prasanth, Alice Egar, Lacee A. Satcher, Samantha Teixeira, Rebekah Levine Coley, Gary Adamkiewicz

**Affiliations:** a Harvard T. H. Chan School of Public Health, 677 Huntington Ave, Boston, MA 02115, USA; b Boston College Department of Sociology, 140 Commonwealth Ave, Chestnut Hill, MA, USA; c Boston College School of Social Work, 140 Commonwealth Ave, Chestnut Hill, MA, USA; d Boston College, Lynch School of Education and Human Development, 140 Commonwealth Ave, Chestnut Hill, MA, USA

**Keywords:** Indoor air quality, PM_2.5_, Public housing, Behavior, Exposure assessment

## Abstract

Fine particulate matter (PM_2.5_) is a ubiquitous air pollutant with known socioeconomic disparities in exposure related to both ambient and indoor sources. Understanding how common indoor behaviors such as smoking, cooking, candle or incense burning, and air freshener use contribute to indoor PM2_.5_ exposure in public housing can inform interventions to protect residents’ health. PM2_._5 data were collected via PurpleAir monitors for one week within 138 units of a single public housing development in New England, United States and corrected via an additive relative humidity model. Study participants completed a pre-monitoring survey and a post-monitoring survey of in-unit PM_2.5_-related behaviors. Participants’ weekly average in-unit PM_2.5_ levels were variable, ranging from 3.6 μg/m^3^ to 142.0 μg/m^3^, with a mean of 19.7 μg/m^3^. Two-thirds (65.9%) of households’ average levels exceeded the EPA annual outdoor standard of 9 μg/m^3^, a heuristic reference given the lack of national indoor guidelines. A multivariable model predicting in-unit average PM2_.5_ based on resident behaviors found significant associations between elevated PM2_._5 and past-week tobacco smoking (121% higher PM2_._5), weekly or more cannabis use (56% higher), and living in an apartment building rather than a townhome (26% higher). Cannabis use, which is studied here for the first time in an uncontrolled residential setting, may represent an underappreciated risk factor for exposure. Interventions directed at mitigating indoor smoking of both tobacco and cannabis may be effective at reducing average in-unit PM_2.5_ exposure in multifamily housing, as compared to combatting other known indoor sources of PM_2.5_.

## 1. Introduction

Indoor air quality (IAQ) has received increasing attention in recent years as a pervasive and persistent environmental health concern, especially in urban residential settings. Fine particulate matter, or PM_2.5_, is a well-studied, ubiquitous form of indoor air pollution with widespread implications for population health [1,2]. PM_2.5_ are solid or liquid particles of varying compositions that are smaller than 2.5 microns in diameter [2]. Exposure to PM_2.5_ is known to cause both short- and long-term negative health outcomes. Short-term effects include irritation of the eyes, throat, and lungs; coughing; difficulty breathing; and asthma aggravation [1,3]. Long-term outcomes include increased risk of premature mortality, adverse birth outcomes, diabetes, cardiovascular disease, and chronic obstructive pulmonary disease [4-6]. Recent evidence also increasingly suggests a link between PM_2.5_ exposure and neurodegenerative disorders [7]. PM_2.5_ exposure is a global health burden: in 2017, it contributed to 4.58 million deaths and 142.52 million years of life lost or with disability [8]. Through the National Ambient Air Quality Standards (NAAQS), the Environmental Protection Agency (EPA) has set the primary (health-based) annual PM_2.5_ ambient standard as 9 μg/m^3^ [9]. However, despite the finding that US residents spend about 90% of their time indoors, there are no established indoor PM_2.5_ standards to protect individuals inside their own homes [10].

Socioeconomic disparities in indoor air pollution exposure, including PM_2.5_, and their links to health inequities have been well-documented [11]. Policies that perpetuate residential segregation, commodify housing, and produce wealth inequity often restrict access to high-quality housing, disproportionately burdening low-income households with hazardous indoor environmental conditions stemming from deteriorating building materials and outdated ventilation systems, for example [12-14]. These conditions increase the risk of PM_2.5_ exposure, among other toxicants, driving disparities in respiratory and cardiovascular health. These health consequences may compound with adverse social exposures like rent burden and housing instability [12, 14]. Those who rent, live in urban settings, or live in neighborhoods with concentrated poverty, are disproportionately exposed to high levels of PM_2.5_ [11,15,16].

While these exposure and health disparities are driven by sociopolitical structures responsible for producing economic and housing quality inequities, local housing policies (e.g., smoke-free policies) and resident behaviors can have a strong direct influence on indoor exposures. For example, previous studies have found that indoor smoking, incense use, candle use, and cooking with insufficient ventilation are associated with elevated indoor PM_2.5_ levels [17-20]. Similarly, interventions to reduce behavioral sources of indoor pollution demonstrated potential to reduce exposures and adverse health outcomes [21]. Though previous studies have used survey-based methods to investigate behavioral influences on IAQ, few have quantified the relationship between resident behaviors and IAQ in low-income housing settings, or independent of building quality, location, and neighborhood context, which may be correlated with resident behaviors and hence bias measured associations [19,21-24].

An emerging and understudied behavioral correlate of poor IAQ is cannabis use. Few studies to date have examined associations between cannabis use and IAQ, in part due to historical criminalization and stigmatization of recreational cannabis use in the US. Cannabis combustion is of increasing research interest as a source of PM_2.5_ due to its recreational legalization in numerous US states, including the study location [25]. Indoor cannabis smoking has been found to produce greater concentrations of PM_2.5_ than tobacco smoking in experimental settings where duration and type of smoking were controlled [26-29]. It also contains additional gas-phase pollutants including free radicals and Volatile Organic Compounds (VOCs) [30,31]. Of additional concern, cannabis smoking has been shown to result in second-hand smoke exposure among non-users sharing indoor environments [32,33]. To our knowledge, the effects of residential cannabis use on indoor PM_2.5_ levels have not yet been quantified in any observational study. Despite evidence that cannabis smoking produces high levels of PM_2.5_, research suggests that both first-hand and second-hand cannabis smoke exposures are perceived as less risky than comparable exposure to tobacco smoke, and the perceived risk of second-hand cannabis smoke is decreasing globally [28,34,35]. These perceptions may influence frequency, duration, and mode of cannabis use (smoking versus ingestion) indoors.

To address limitations in the extant literature, this study sought to investigate factors shaping in-unit PM_2.5_ among a large sample of urban low-income households living within a single, large public housing development in New England. At the time of data collection, demolition and redevelopment of the complex was scheduled after decades of underinvestment and deferred maintenance, but had not yet begun. The redevelopment plan called for increasing energy efficiency and environmental quality, for example via improved ventilation in new units. Previous work found that redevelopment of conventional public housing into green public housing in the same municipality as this study was associated with a 57% decrease in average in-unit PM_2.5_ [36]. While building design and building-level policies are the primary sites of intervention rather than individual-level factors, understanding the contributions of occupant behaviors to in-unit PM_2.5_ levels may provide additional insight that households and housing management can leverage to manage IAQ and health risks both individually and collectively. Identifying behaviors affecting indoor air quality has the potential to inform communication and education among residents and building management.

Given the value of leveraging indoor PM_2.5_ sources as potential intervention targets, our objective was to explore associations between resident behaviors and in-unit PM_2.5_ concentrations among units of a single public housing complex. Specifically, we evaluated associations between average in-unit PM_2.5_ concentrations and self-reported behaviors expected to elevate PM_2.5_ (in-unit tobacco smoking, air freshener use, candle use, incense use, cooking, and general cannabis use) and those expected to lower PM_2.5_(window opening).

## 2. Materials and methods

### 2.1. Study design

**Fig. 1.**
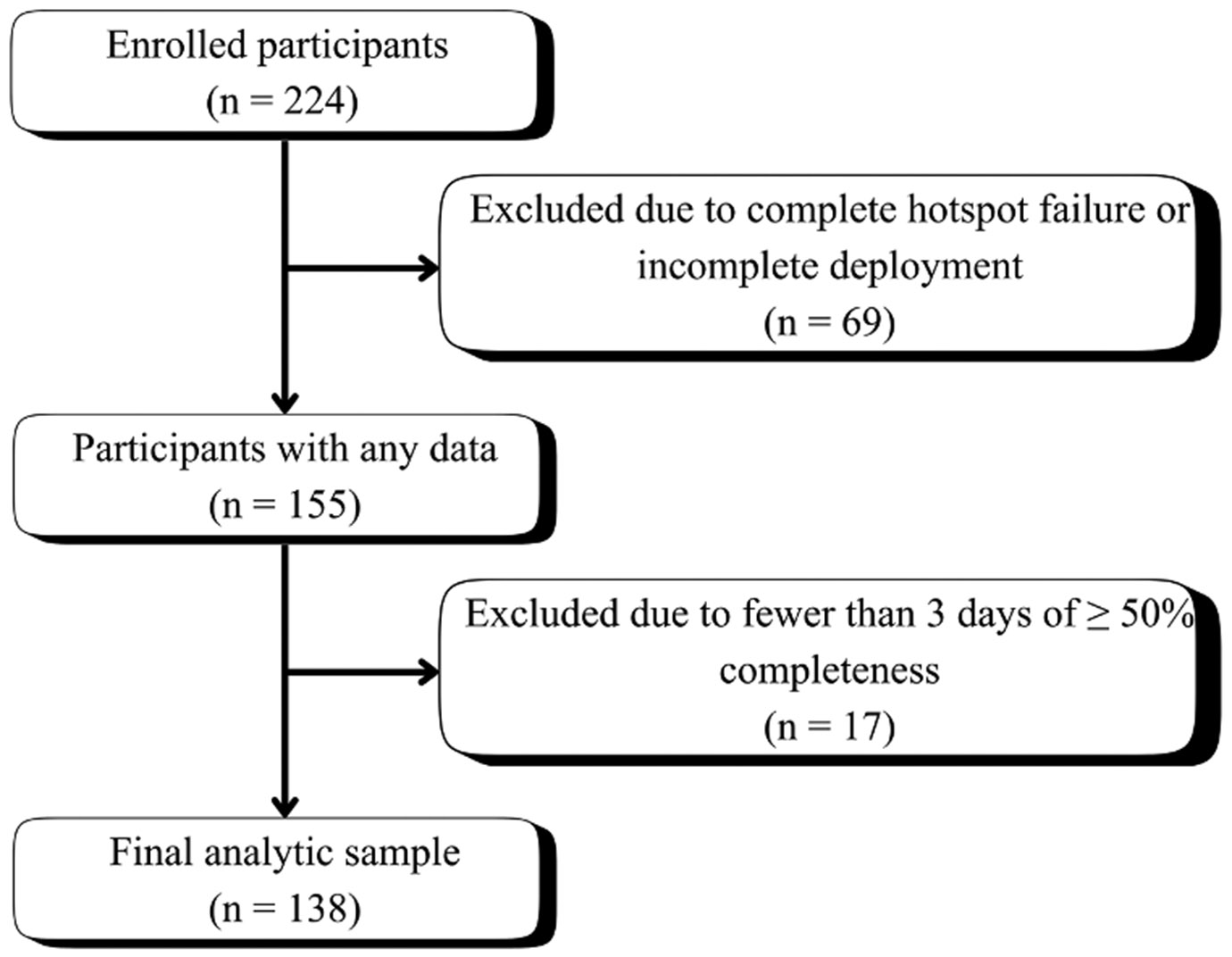
Flow chart of participant inclusion and exclusion criteria applied to 224 enrolled participants, resulting in an analytic sample of 138 participants.

The Housing Opportunity and Mobility Experiment (HOME) Study is a longitudinal project following approximately 450 households of a racially and ethnically diverse public housing development in New England, United States, through its redevelopment into mixed-income housing. The community, built in the 1930s, is comprised primarily of three- to four-floor multi-unit apartment buildings with shared entrances, along with a smaller number of attached multi-story townhomes with larger units and individual entrances but shared internal walls. Data reported here were drawn from baseline data collection conducted between October 2022 and May 2023, prior to initiation of the demolition and redevelopment process. This study focuses on a subsample of the HOME study population with data on our targeted constructs. All adult HOME participants completed an in-person pre-monitoring survey inclduing demographic, environmental, health, and behavioral topics. Survey respondents were offered optional participation in environmental monitoring of their units for the subsequent week, followed by an additional brief post-monitoring survey on in-unit behaviors. A total of 224 adults participated in the environmental monitoring and post-monitoring survey, with 138 retained for analysis after excluding participants with incomplete monitoring data (see Fig. 1). All procedures were performed in compliance with relevant laws and institutional guidelines and were approved by the Institutional Review Board (IRB) of Boston College under reference number 21.266.01 on 05/07/2021. The privacy rights of human subjects were observed and informed consent was obtained prior to data collection.

### 2.2. Behavioral data

Most behavioral variables (cooking, tobacco smoking, candle use, incense use, air freshener use, and window opening) were drawn from the brief environmental exposure questionnaire administered at the conclusion of air quality monitoring, which captured the frequency of behavioral engagement during the sampling period (Table 1). An additional question was drawn from the broader pre-monitoring survey about marijuana use, regardless of whether it was smoked or consumed, in the past 12 months (Table 1).

Responses to the post-monitoring questionnaire were dichotomously coded as “yes” if the household self-reported engagement in the activity at least once within the week of environmental monitoring, otherwise as “no”. Cannabis use was dichotomously coded as “yes” for responses of ‘Once a week or more’ and “Nearly every day”, otherwise coded as “no” to match the weekly scale of the post-monitoring questionnaire.

### 2.3. Air quality data

Environmental data collection was conducted via PurpleAir Classic Air Quality Monitors in the main living space (typically the living room) of participants’ units. Devices were connected to Orbic Verizon Speed Mobile Hotspots to report data remotely. The PurpleAir devices collected relative humidity (RH), temperature, pressure, and PM_2.5_ data at 2-minute intervals. Ambient (outdoor) PM_2.5_ data was collected by EPA monitor via Teledyne T640 at 5.0 LPM with Network Data Alignment. The EPA monitoring site was located between 0.05 and 0.5 miles from the housing complex residences. Participant-specific ambient PM_2.5_ was calculated by time-matching outdoor concentrations to each participant's indoor sampling period and averaging these outdoor values to generagte a single ambient estimate per participant, which was included in the models to adjust for outdoor influence. Concurrent in-unit PM_2.5_ data was collected by PurpleAir devices via two laser particle sensors (Plantower PMS5003), which are referred to as channel A and channel B. While the PurpleAir device has been shown to be precise, its accuracy is inadequate [37,38]. Previous work has found the PurpleAir PM_2.5_ package to overestimate raw PM_2.5_ levels by around 40% in most parts of the United States, under both ambient and smoke conditions, though measurements can vary by region [37]. In the absence of a municipal-specific PurpleAir correction method, and after extensive literature review, two published correction methods, one US-wide and one regional, were considered for use in this study due to their spatial proximity to the study site and their deployment of identical low-cost sensors.

Our evaluation of these two correction methods is included in the Supplemental materials (Text S1). We selected the US-wide method, which is an additive RH correction model developed from almost 12,000 24-h average PurpleAir PM_2.5_ measurements collocated with Federal Reference Method (FRM) or Federal Equivalent Method (FEM) across 16 states in the US [37]. This quality assurance and quality control (QA/QC) procedure averages daily, excludes periods below 90% completeness, and excludes individual data points with largely disagreeing channel A and B values prior to application of an additive RH model using onboard RH measurements (Eq. (1)). This US-wide method was found to reduce the RMSE of the daily averaged PM_2.5_ data from 8 to 3 μg/m^3^ across the US when compared to regulatory measurements [37]. Of note, the US-wide method was developed using outdoor PurpleAir measurements which differ from the indoor setting of this study.

Eq. (1). Additive RH correction model developed by Barkjohn et al., 2021, applied to all PurpleAir PM_2.5_ measurements.


$${\text{PM}}_{2.5}=0.524\times {\text{PA}}_{\text{cf}\_1}-0.0862\;x\;\text{RH}+5.75$$

Real-time PurpleAir data were retrieved via Python 3.11.1 and the PurpleAir RESTful API (https://api.purpleair.com/). Data were trimmed to 30 minutes post sample start time and 30 minutes prior to sample end time to remove unreliable data due to device set up and removal processes. As a result of poor performance from the Orbic Verizon Speed Mobile Hotspots, data loss was higher than expected. Individual data points were excluded consistant with recommendations of Barkjohn et al., but to preserve data for analyses, the daily completeness threshold was lowered to 50% informed by sensitivity analyses (Table S1) [37]. Participants were excluded from the study if they had fewer than three total days of data that met quality control standards before a full-sample arithmetic mean was calculated, referred to as the weekly in-unit average. A total of 138 participants were retained for analysis (Fig. 1). Data cleaning and correction was completed in PyCharm (version 2024.3.6) with interpreter Python 3.11.1.

### 2.4. Statistical analysis

For statistical analyses, PM_2.5_ values were natural log-transformed to meet data normality requirements. As missing data can bias samples and limit statistical power, we imputed missing data on behavioral and demographic variables. Counts and percents of missing values for selected sociodemographic and behavioral variables are shown in the Note. of Table 2 and in Table 3. Multiple imputation was applied to the analytic sample based on Fully Conditional Specification using the Multivariate Imputations by Chained Equations (MICE) algorithm to impute missing values of behavioral variables, using the full range of response categories (i.e., before dichotomization) for more precise imputation [39]. Several sociodemographic, behavioral, and environmental variables were included in the imputation model, along with the previously listed behaviors, building type (townhome versus apartment) and number of bedrooms—both obtained from administrative data sources—as well as in-unit log-transformed PM_2.5_ and outdoor PM_2.5_. Survey questions and response categories for all survey variables included in the imputation model are listed in Table S2.

Predictive mean matching was used to impute missing values for continuous variables (age, household size, and monthly income); proportional odds modeling was used to impute ordered categorical variables (education, cooking, incense, smoking, candles, e-cigarettes, cannabis, home too hot, home too cold, air conditioning, air freshener, and window-opening); and polytomous logistic regression was used to impute unordered categorical variables (ethnoracial group). This was repeated to derive 30 total imputed datasets. Variation in the mean and standard deviation of imputed values for ordinal behavioral variables across iterations is shown in Fig. S1, suggesting adequate MICE algorithm convergence [40]. Imputation diagnostics for each main model variable are shown in Table S3. Values of lambda ranged from 0.01 to 0.05, suggesting relatively low proportions of total variance attributable to missingness, while Relative Increase in Variance (RIV) ranged from 0.01 to 0.06, again suggesting minimal impacts of missingness on variance (Table S3) [41].

We fitted multivariable linear regression models to each imputed dataset and pooled coefficients and standard errors using Rubin’s rules to estimate adjusted associations between each behavioral variable and log-transformed average in-unit PM_2.5_ concentrations, while accounting for imputation uncertainty. Binary in-unit tobacco smoking, air freshener use, candle use, cooking, incense use, and window opening within the past week, as well as general cannabis use once a week or more within the past year, were included as predictors in the main model. Additionally, the model adjusted for unit type (townhome versus apartment) and ambient average PM_2.5_. Several demographic and building variables were considered for inclusion as confounders in the model, based on prior evidence of correlations with indoor PM_2.5_ [11, 13,17-19,23,24,26]. Of these variables, age, education status, primary language, gender, race/ethnicity, income, number of inhabitants, number of bedrooms, and tenure at the housing complex were not meaningfully associated with PM_2.5_ when included all together in a multivariable linear model alongside unit type, outdoor PM_2.5_, and the behaviors listed in Table 3, nor did their inclusion materially affect effect estimates of primary behaviors. Inclusion of these variables resulted in model oversaturation, and they were thus excluded from subsequent analyses in favor of a parsimonious final model.

All analyses, including demographic counts, behavioral counts, PM_2.5_ distributions, univariable linear regressions, and multivariable linear regressions, were conducted in RStudio (version 2024.12) with R 4.4.1 or in PyCharm (version 2024.3.6) with interpreter Python 3.11.1. Figures were created in PyCharm (version 2025.1.3.1) with interpreter Python 3.13 or in Canva.

## 3. Results

### 3.1. Participant demographics and participation in IAQ-related behaviors

Participant demographic characteristics are summarized in Table 2. Respondents were mostly women (78%), half identified as Hispanic/Latino (50%), and the mean monthly income was $1913. Data on behaviors related to in-unit air quality are displayed in Table 3. Within the sampling week, 80% reported in-unit cooking activity, 18% reported in-unit tobacco smoking, 22% reported in-unit candle use, 17% reported in-unit incense use, 43% reported in-unit air freshener use, and 72% reported opening a window. Meanwhile, 12% of respondents reported using cannabis once a week or more within the past year.

### 3.2. In-unit PM_2.5_ concentrations

**Fig. 2.**
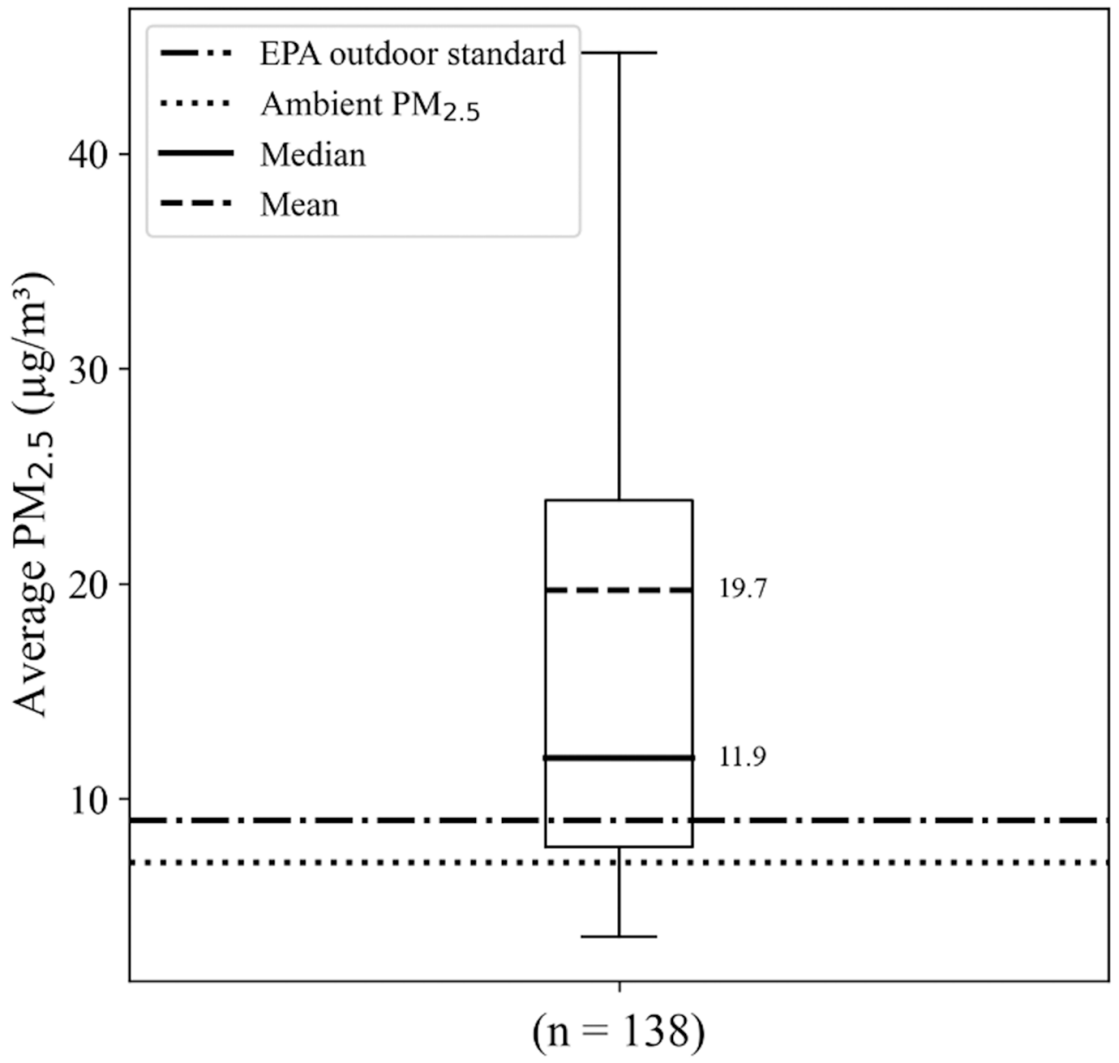
Distribution of corrected weekly average in-unit PM_2.5_ concentrations (μg/m^3^) across study participants^a^.

Corrected weekly average PM_2.5_ concentrations ranged from a minimum of 3.58 μg/m^3^ to a maximum of 142.03 μg/m^3^ with a median of 11.89 μg/m^3^, a mean of 19.70 μg/m^3^, and a standard deviation of 19.72 μg/m^3^ (Fig. 2). Almost two-thirds of the sample (65.9%) had average in-unit PM_2.5_ levels greater than the EPA annual outdoor standard of 9 μg/m^3^, even though the overall ambient average PM_2.5_ across all sampling periods, 7.03 μg/m^3^, was below this standard.

### 3.3. Sensitivity analyses of PM_2.5_ correction procedures

Sensitivity analyses were conducted comparing a) the main model using imputed US-wide corrected HOME study data, b) the uncorrected data, and c) the unimputed data, showing similar effect estimates, 95% CIs, and p-values across all included variables (Table S4). Sensitivity analyses on QA/QC procedures were conducted using daily completeness thresholds of 60%, 70%, 80%, and 90%. Data sets of varying daily completeness yielded effect estimates that were directionally consistent with the primary model (daily completeness threshold of 50%). Key associations, including tobacco smoking, cannabis use, and unit type retained highly similar effect estimates across all daily completeness thresholds. Tobacco smoking remained significant across all thresholds, while cannabis and unit type became borderline significant with widening CIs when n = 66, likely driven by the dramatically lower sample size. No effect reversals were observed across all variables (Table S1).

### 3.4. Modeling in-unit PM_2.5_

First, we examined the independent contribution of each behavioral variable to average in-unit PM_2.5_. Results of univariable linear regression models with log-transformed in-unit average PM_2.5_ as the outcome, to singly consider each behavioral factor as well as unit type and participant-unique outdoor average PM_2.5_, are summarized in Table 4.

Next, we fit a multivariable linear regression to assess the relative contribution of each behavioral variable to average in-unit PM_2.5_ when adjusting for other all other behavioral variables, building type, and ambient PM_2.5_ (Table 5). Correlations between model predictors, shown in Table S5, ranged from −0.156–0.313, suggesting low to moderate correlations and a lack of collinearity between model variables.

Cooking, window opening, and ambient PM_2.5_ were not significantly associated with average in-unit PM_2.5_ in univariable or multivariable models ( Tables 4 and 5). Meanwhile, candle, incense, and air freshener use were all significant (p < 0.05) predictors in univariable models, associated with a 36.3% (95% CI [2.0–82.1%]), 49.2% (95% CI [9.8–102.8%]), and 38.9% (95% CI [8.8–77.5%]) increase in average in-unit PM_2.5_, respectively, but were not significantly associated with PM_2.5_ in the multivariable model. Living in a townhome as opposed to in an apartment building approached significance in association with average in-unit PM_2.5_ in the univariable model (p = 0.060) and was significantly associated with 25.7% (95% CI [2.4–43.4%]) lower average in-unit PM_2.5_ in the multivariable model (p = 0.035) (Tables 4 and 5).

In the univariable linear regression model, self-reported in-unit tobacco smoking within the past week was associated with a 156.3% (95% CI [95.6–236.0%]) increase in average in-unit PM_2.5_ levels (p < 0.001). This effect was slightly smaller yet still strongly significant in the multivariable model, in which in-unit tobacco smoking in the past week was associated with a 120.7% (95% CI [63.3–198.2%]) increase in average in-unit PM_2.5_ (p < 0.001), after adjusting for other behavioral variables, unit type, and outdoor PM_2.5_. As in-unit PM_2.5_ was modeled on the log scale to address data normality concerns, these estimates correspond to multiplicative effects; for example, if a non-smoking unit had an average PM_2.5_ level of 10 μg/m^3^, a smoking unit would be expected to have an average level of 22.1 μg/m^3^, all else held constant. Finally, self-reported cannabis use (once a week or more within the past year) was significantly associated with a 71.1% (95% CI [18.5–147.0%]) increase in average in-unit PM_2.5_ (p = 0.005) in the univariable model and with a slightly attenuated but still strongly significant 58.5% (95% CI [14.3–119.9]) increase in average in-unit PM_2.5_ (p = 0.007) in the multivariable model (Tables 4 and 5).

### 3.5. Tobacco-cannabis interaction

**Fig. 3.**
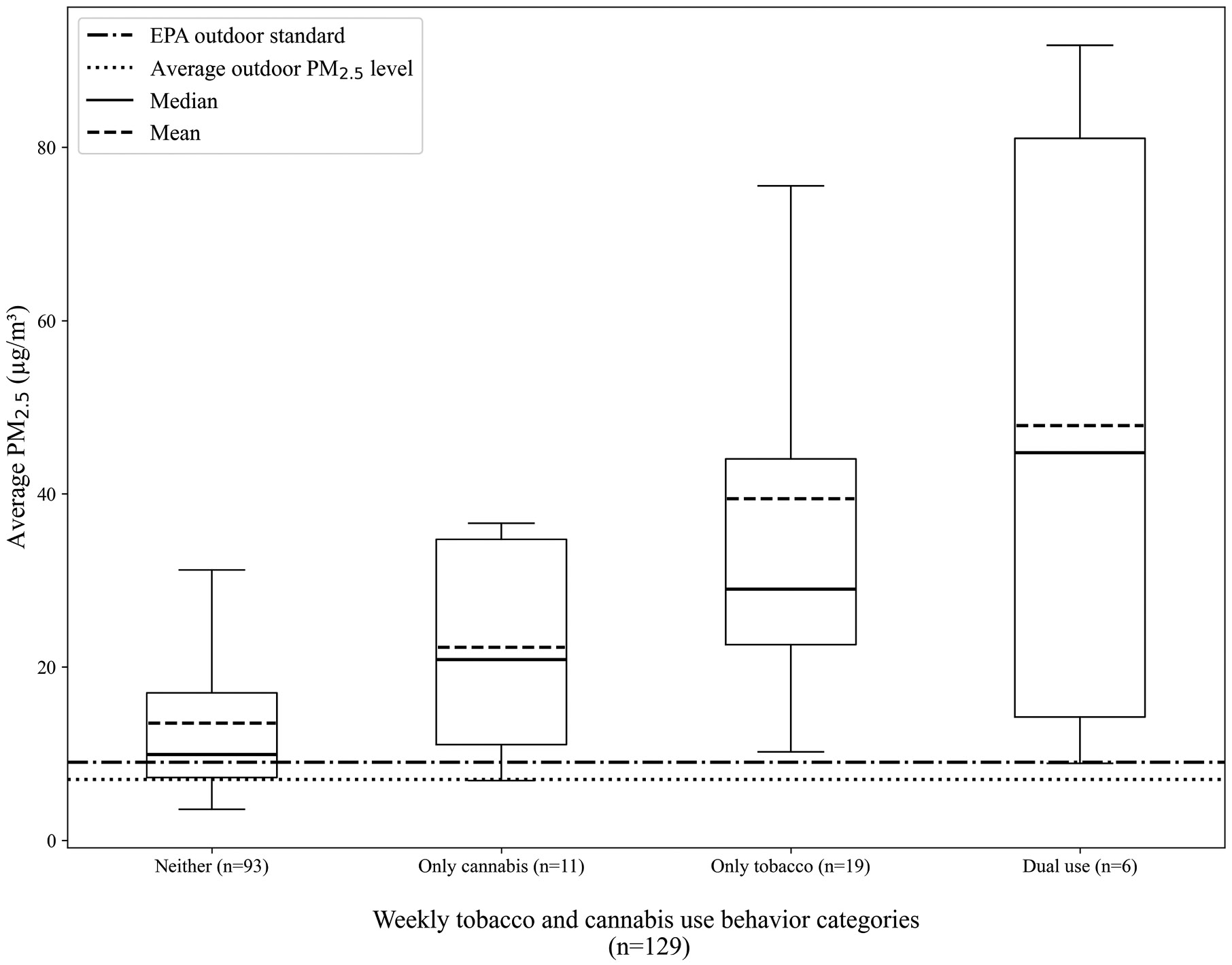
Distributions of average in-unit PM_2.5_ concentrations (μg/m^3^) across observed tobacco and cannabis use behavior categories: neither past-week tobacco smoking nor weekly or more cannabis use (n = 93); only cannabis use (n = 11); only tobacco smoking (n = 19); and both tobacco smoking and cannabis use (n = 6) using unimputed data. The dual use category has a notably small sample size and therefore should be interpreted with caution^b^.

Given the statistical importance of both tobacco and cannabis use revealed in our multivariable model, we examined descriptive bivariate associations in the distributions of average PM_2.5_ levels across substance use patterns —participants who reported neither, only cannabis use, only tobacco smoking, or both—using purely observational data (without imputation). Analyses revealed variation in the distributions of average in-unit PM_2.5_ across households with different tobacco and cannabis use behavior patterns (Fig. 3). Residents who reported neither past-week in-unit tobacco smoking nor cannabis use exhibited the lowest average in-unit PM_2.5_ concentrations, with the lowest central tendency and upper-range values (median = 9.9 μg/m^3^, mean = 13.5 μg/m^3^, 75th percentile = 17.0 μg/m^3^, upper whisker = 31.2 μg/m^3^) (Fig. 3). Residents who reported only cannabis use had lower central tendency and upper-range values (median = 20.8 μg/m^3^, mean = 22.3 μg/m^3^, 75th percentile = 34.8 μg/m^3^, upper whisker = 36.6 μg/m^3^) than those who reported only past-week tobacco smoking (median = 29.0 μg/m^3^, mean =39.4 μg/m^3^^3^, 75th percentile = 44.0 μg/m^3^, upper whisker = 75.5 μg/m^3^). While the sample size was very small (n = 6), residents reporting both past-week tobacco smoking and weekly or more cannabis use had the highest central tendency and upper-range PM_2.5_ concentrations (median = 44.7 μg/m^3^, mean = 47.9 μg/m^3^, 75th percentile = 81.0 μg/m^3^, upper whisker = 91.8 μg/m^3^). These results indicate a positive pattern between the number of possible smoking behaviors reported and PM_2.5_, although the dual use category is too small to draw firm conclusions.

In a subsequent analysis, we re-estimated the multivariable model adding an interaction term between tobacco smoking and cannabis use, given the potential additive effect of these behaviors suggested by descriptive analyses (Fig. 3). However, we observed no significant interaction between tobacco smoking and cannabis use (p = 0.329) when adjusting for all other behavioral variables, unit type, and ambient PM_2.5_ (Table 6). Patterns of all other predictors remained consistent with the main effects model (Table 5).

## 4. Discussion

Indoor PM_2.5_ concentration is determined by a variety of factors, including infiltration from outdoors, building ventilation, and resident behaviors. Given that building characteristics are costly to change and, in the context of the housing complex under study, already slated for redesign to improve building quality and health, understanding the contribution of resident behaviors to indoor PM_2.5_ may help inform interventions to reduce indoor PM_2.5_ exposure in similar housing contexts and to supplement ongoing redevelopment efforts. The use of tobacco, cannabis, candles, and incense, as well as cooking, have been previously shown to produce PM_2.5_ [18,19,27]. Conversely, opening windows can decrease indoor PM_2.5_ concentrations if they exceed ambient concentrations [22]. While the influence of tobacco, cannabis, candles, incense, cooking, and window use on PM_2.5_ has been studied in controlled settings, their associations with indoor PM_2.5_ have rarely been examined in observational settings with low variation in building characteristics and geographic location. To our knowledge, this is the first such study conducted within a single housing complex, a design that is methodologically important to isolate unique behavioral contributors above and beyond built and natural environmental features. In this work, we found in-unit tobacco smoking and cannabis use to be the strongest behavioral predictors of elevated PM_2.5_, while the effects of other studied behaviors were likely attenuated by low frequency of use and the week-long PM_2.5_ averaging period.

### 4.1. Resident behaviors

#### 4.1.1. Tobacco use

Of the examined behavioral variables, in-unit tobacco smoking was associated with the largest increase in average in-unit PM_2.5_ levels, 120.7% (p < 0.001), after adjusting for confounding variables (Table 5). This result is in line with previous findings that indoor tobacco smoking increases PM_2.5_ levels in residential units [19]. In this study, tobacco smoking may have been under-reported due to the community’s long-standing no-smoking policy and the self-reported nature of all behavioral data. Although a previous study validated the accuracy of self-reported smoking behaviors in subsidized housing, it was not conducted in the context of a non-smoking policy [42]. Previous studies have shown trends of underestimation via self-report depending on the population [43]. In the presence of potential enforcement consequences, our study participants may have been incentivized to underreport in-unit smoking. However, as of the most recent publicly available report, no residents across the housing authority’s broader portfolio had been evicted soley due to indoor tobacco smoking [44]. The absence of evictions does not preclude other forms of enforcement, but it does suggest that severe consequences may be uncommon. As underreporting would bias associations towards the null, our results should be interpreted as lower-bound estimates of the true effect.

#### 4.1.2. Cannabis use

In this real-world, uncontrolled residential setting, weekly or more frequent cannabis use was associated with a 58.5% increase in average in-unit PM_2.5_ after accounting for other modeled behaviors—including cooking, tobacco smoking, candle, incense, air freshener, cannabis, and window use—as well as unit type and ambient PM_2.5_ levels (Table 5). Although the effects of cannabis smoking on PM_2.5_ have been increasingly well characterized in controlled experimental environments—where smoking duration and methods are standardized—the effect of uncontrolled cannabis use in real-world settings has not, to our knowledge, been previously studied [26-29]. Our findings highlight the importance of such research. The pre-monitoring survey question on which these results are based—“In the past 12 months, how often did you use marijuana, which is also called pot or weed?”—does not identify mode (inhalation versus ingestion) nor location (indoors versus outdoors) of consumption, as the question was designed to characterize general substance use. Mode of cannabis consumption is an important consideration, as edible cannabis use is not expected to contribute to PM_2.5_ levels, while previous evidence suggests that both smoking and vaping behavior elevate PM_2.5_ [27]. Without direct mode or location information, our results likely represent lower-bound estimates of the true effect of indoor cannabis smoking. However, we maintain the validity of our result as national data suggest that approximately 80% of adult cannabis users smoke it and anecdotal evidence from HOME interviewer notes indicates frequent observations of cannabis odor in hallways and signs of cannabis smoking within individual units [45]. We recommend an improved measure of in-unit cannabis smoking. In future work, we are implementing a cannabis survey question designed from an IAQ perspective to more accurately capture both the location and mode of cannabis use.

As the temporal difference—within the past year versus within the past week—between the cannabis use and other behavioral questions could be of concern for correct cannabis use classification, only responses of “Once a week or more” and “Nearly every day” in the past year were categorized as positive for cannabis use. While this could introduce misclassification, it is unlikely given that cannabis is often used on a recurring basis. Findings from cannabis use trends reported in the 2022 US National Survey on Drug Use and Health indicate that cannabis users are disproportionately high-frequency users, as 42.3% of those who reported any past-month use also reported use on 21 or more days, a pattern repeated across subpopulations [46,47]. These results support our assumption that those who report use of cannabis at least once a week on a yearly scale likely partook within our sampling week . Additionally, the pre-monitoring survey was administered immediately preceding environmental monitoring, further reducing potential misclassification due to recall bias towards recent use.

The 12% self-reported rate (Table 3) and observable association with PM_2.5_ adds to the evidence on growing rates of adult cannabis use and to evidence regarding the potential for negative health effects [25]. These results suggest that cannabis use may have both individual and shared effects on health through indoor air pollutants. Our findings indicate a need for greater policy attention to limit indoor cannabis smoking. The HOME community's current resident-facing Non-Smoking Policy is ambiguous regarding cannabis. The policy disallows smoking, defined as lighted tobacco products, and fails to explicitly include cannabis products [44]. Cannabis-related terms were not included in the Non-Smoking Policy lease addendum, nor a resident engagement campaign designed to educate residents about the policy [44,48]. As all indoor smoking is prohibited under the current policy, we recommend explicit inclusion of “smoked cannabis or marijuana products” in all resident-facing definitions of smoking and secondhand smoke, as well as inclusion of cannabis-related terms (e.g. joint, weed), to improve policy understanding and compliance.

#### 4.1.3. Interaction between tobacco and cannabis use

As tobacco and cannabis use were the only two clearly significant behavioral contributors to average in-unit PM_2.5_ concentrations among our participants, we expected PM_2.5_ exposure among non-smokers to be similar to that of outdoor levels. However, only 27% of non-smoking households had PM_2.5_ concentrations at or below the average outdoor level across all sampling periods of 7.03 μg/m^3^. This difference could be attributed to the cumulative effect of the remaining PM_2.5_ sources (incense, cooking, candles, and air fresheners) that are known to release PM_2.5_, other unmeasured factors, and infiltration of PM_2.5_ from neighboring units. Infiltration is a known pathway that elevates PM_2.5_ in multifamily housing; the discepancy between in-unit concentrations in non-smoking households and outdoors indicates the necessity of collectively reducing all kinds of indoor smoking in multi-unit housing [13]. It also, unfortunately, highlights the limited autonomy of non-smoking residents when facing exposure to indoor air pollution, especially when living in units with limited ventilation options. Unsurprisingly, in descriptive analyses, households that reported both tobacco smoking and cannabis use had higher PM_2.5_ levels than those using just one or neither of these substances (Fig. 3). However, we did not find evidence of a significant interaction between tobacco and cannabis use in our supplementary analysis (Table 6). This null finding may reflect insufficient power to detect effect modification, given the modest sample size and the very low prevalence of concurrent tobacco and cannabis use. The absence of a statistically significant interaction effect should therefore be interpreted cautiously and does not imply the absence of combined or additive impacts. Future research with larger sample sizes and more accurate measurement of in-unit cannabis smoking is needed. Research should also explore the extent to which behavioral contributors to PM_2.5_ may be cumulative, with a greater number of sources leading to higher indoor air pollution levels. Additionally, our sample size precluded subgroup analyses, but future research may benefit from stratifying by demographic and contextual strata.

#### 4.1.4. Air fresheners

Air fresheners were significantly associated with a 38.9% increase in PM_2.5_ in the univariable model (Table 4), but contrary to our expectations, we did not find a significant association between air freshener use and average in-unit PM_2.5_ concentrations in the multivariable model (Table 5). Although aerosol air fresheners are a known source of PM_2.5_, their particle release is typically brief, restricted to specific spaces, and thus less likely to exert a strong influence on weekly average PM_2.5_ levels in a home compared to more prolonged and pervasive activities like smoking [49]. Furthermore, it is important to note that air fresheners are more frequently associated with an increase in VOCs than PM_2.5_, so households in which both air freshener use and indoor smoking occur are likely to experience elevated exposure to multiple air quality hazards [49,50].

#### 4.1.5. Candles and incense

Similarly, both candles and incense were significantly associated with an increase in PM_2.5_ in the univariable model, 36.3% and 49.2% respectively (Table 4), but neither remained significant after accounting for other variables (Table 5). These null findings may be a result of low statistical power given the relatively low reported use of incense (17%) and candles (22%) (Table 3), as well as their low frequency of use. Despite previous findings that candle-burning emits PM_2.5_, our model did not identify this behavior as a significant predictor of average PM_2.5_ levels [18]. It is known that the type of burning activity (i.e., whether a candle is burned steadily or is blown out and relit frequently) and type of candle wax impact PM_2.5_ emissions [51,52]. Our survey design did not specify either of these factors, which may have influenced the observed effect of candle use on PM_2.5_ levels in residential buildings. A narrower definition of candle use, such as number of blowout events, may have a larger impact on PM_2.5_ concentrations in this setting. Most likely, our results were also influenced by the low reported frequency of candle use within our study population: 70% of those who reported candle use indicated the lowest frequency use option of 1–3 days. Previous work suggests that PM_2.5_ emitted from candles decays within only a few hours after extinguishment, which may have further limited our ability to detect an effect on weekly average levels, as opposed to hourly or peak levels, which were unable to be evaluated given PurpleAir data reliability constraints [17,37]. Likewise, our multivariable model did not find incense use to be significantly associated with PM_2.5_ despite previous findings that incense burning releases particulate matter [18,20]. This finding may again be attributed to the infrequent use of incense among users, as 62.5% of incense-using households reported the lowest use category of 1–3 days, or to the ephemeral nature of incense PM_2.5_ emissions [19,53]. Thus, eliminating infrequently used and transient sources of PM_2.5_, in this context candles and incense, may not be a top priority when trying to reduce residents’ long-term average exposure levels.

#### 4.1.6. Cooking

Previous studies have identified cooking as one of the largest contributors to average indoor PM_2.5_ levels [54,55]. Surprisingly, cooking activity was not significantly associated with PM_2.5_ levels in either our univariable (Table 4) or multivariable models (Table 5) even though 80% of study participants reported cooking at least once within the monitoring week (Table 2). The study units were outfitted with electric stoves, which are associated with lower PM_2.5_ emissions during cooking than natural gas stoves [56]. Though cooking would be expected to produce PM_2.5_ regardless of stove type, the use of electric stoves coupled with the low temporal resolution of our data may explain this null finding [57]. This result could also be related to the high proportion of participants who opened their windows (72%), the lack of distinction between frequent and infrequent cooking, or the lack of differentiation between types of cooking activity, as some forms of cooking are known to produce far greater amounts of PM_2.5_ than others [58].

#### 4.1.7. Window-opening

Window-opening behavior was also non-significant in our study population. Many studies examining the link between window-opening behavior and indoor air pollution focus on the infiltration of outdoor-origin PM_2.5_ into the residence, rather than the expulsion of indoor-origin PM_2.5_ from the residence [59-61]. Previous research modeling particle infiltration in US homes has found window-opening behavior to be associated with only minor impacts on infiltration of outdoor PM_2.5_ [61]. One recent local study observed that PM_2.5_ of outdoor origin in naturally ventilated homes increases in the summer relative to the winter; however, researchers did not measure the flow of indoor PM_2.5_ into the outdoor environment [59]. Studies showing a beneficial impact of window opening on IAQ are largely restricted to upper-level units [62]. We were unable to account for unit level in our analyses.

To our knowledge, no studies have been conducted to determine correlations among window-opening behavior and indoor PM_2.5_ level within residential buildings in the Northeast US. This region generally has low outdoor PM_2.5_ levels [63,64]. Our null window-opening finding, despite low ambient levels concurrent with high in-unit concentrations, may reflect the outsized influence of behavioral factors such as tobacco and cannabis use or the lack of adjustment in our model for unit level, and warratns further investigation.

### 4.2. Unit type

In the multivariable model, living in a townhome was significantly associated with 25.7% lower PM_2.5_ levels compared to living in an apartment. Given that the townhome units included in our study have independent entrances and are larger relative to apartments, pathways for air movement between units, and thus infiltration from neighboring units, are far more limited. This trend aligns with previous findings that renters in multifamily housing experience higher PM_2.5_ concentrations than individuals in other housing configurations [15]. Furthermore, the spread of secondhand smoke into common areas and non-smoking units of multi-unit low-income housing has been frequently identified as a health concern [65,66]. Improved ventilation to the outdoors and greater insulation between units may help alleviate infiltration from neighboring units in similar high-density housing.

### 4.3. Limitations

Several limitations constrain the interpretability of these results. First, the US-wide method used to correct PM_2.5_ data was developed using ambient PM_2.5_ data sets and thus its performance may be worse in indoor environments. Despite this, the US-wide method was found to substantially increase the accuracy of indoor PurpleAir PM_2.5_ data in a Baltimore-based study of 151 homes and to outperform the correction method created from this indoor Baltimore data set in our New England-based validation study (Text S1) [67]. Second, reviewed PurpleAir correction methods perform worse when PM_2.5_ concentrations are higher than 30 μg/m^3^, which was the case for 18.4% of our study households, as the training data was primarily comprised of lower concentrations [37,67]. However, the US-wide method outperformed the Baltimore method even for homes with high weekly mean levels [37]. We recommend further investigation of PurpleAir-specific correction methods above 30 μg/m^3^ as existing approaches are limited below this threshold [37,67]. Third, the US-wide method data set was predominantly comprised of urban sites, which is applicable to our study site, but regions distant from New England including Iowa and California were overrepresented [37]. Fourth, our data set had a high level of missing PM_2.5_ data; however, the final analytical sample of 138 remains relatively large for an intensive in-home human subjects study. Efforts were made to retain reliable data without compromising data integrity, including lowering the daily completeness threshold which the publishing authors recognized as strict [37]. This decision was supported by sensitivity analyses (Table S1) which found similar point estimates and no effect reversals between the most strict and most lenient daily completeness thresholds. Additionally, the employed correction method relied on daily averages, which limited our ability to analyze short-term peaks in PM_2.5_ concentrations, and hence may have masked the effects of activities such as cooking, burning candles, and burning incense. We suspect that these activities are significant contributors to peak PM_2.5_ concentrations. As previously noted, our ability to identify behaviors such as candle and incense use as significant contributors was also limited by the low reported frequency of these behaviors among study participants. Further, the self-reported behavioral data may be unreliable, particularly regarding in-unit tobacco use as it is prohibited, even though self-reporting has been previously validated [42].

## 5. Conclusions

PM_2.5_ was measured for one week in 138 units of a single large public housing complex in New England, United States. Two-thirds of households’ average in-unit PM_2.5_ concentrations exceeded the EPA annual outdoor standard of 9 μg/m^3^, used here as a benchmark in the absence of a national indoor standard. PM_2.5_ measurements combined with resident survey data revealed that behaviors of in-unit tobacco smoking and cannabis use were strongly associated with elevated average in-unit PM_2.5_ concentrations. Residents reporting in-unit tobacco smoking in the past week had 121% higher PM_2.5_ averages than residents reporting no IAQuse after adjusting for behaviors—including cooking, candle, incense, air freshener, cannabis, and window use—as well as unit type and ambient levels. Using cannabis once a week or more was associated with 59% higher PM_2.5_ in the adjusted model, which is a novel finding in an uncontrolled residential setting. In contrast, behavioral factors that have previously been associated with higher PM_2.5_—such as use of air fresheners, incense, candles, cooking, and not opening windows—were not significantly associated with weekly average PM_2.5_. Ambient PM_2.5_ was also not significantly associated with average in-unit PM_2.5_. A physical building factor, unit type, was significantly associated with PM_2.5_ concentration; residents in townhomes had, on average, 26% lower PM_2.5_ than residents in high-density apartment buildings. This study highlights high average exposure to the indoor air pollutant PM_2.5_ among residents of a low-income public housing complex. This observed exposure is not direct evidence of health effects, though exposure to PM_2.5_ is a known health risk [1-7].

To address this exposure, potential interventions can be co-developed with residents, such as community education programs focusing on the impact of indoor smoking on indoor air quality and health outcomes, together with support for connecting residents to cessation programs [68]. Interventions promoting resident access to cessation programs via transportation assistance, incentives for participation, and on-site peer support groups may support residents in reducing tobacco smoking and resultingly improve IAQ. Despite development-wide policies prohibiting indoor smoking, nearly one in five participants reported in-unit tobacco smoking in their unit during the one-week sampling period. Educational efforts including flyers, presentations, and community discussions can raise awareness of both the individual and collective health impacts of secondhand smoke and encourage a behavioral shift from indoor to outdoor smoking, as one way of reducing harm. These initiatives should also communicate the potentially comparable risk of indoor cannabis smoking with respect to PM_2.5_ exposure, especially given the perception that cannabis smoke is less dangerous [26,28,34]. We further recommend explicit mention of smoked cannabis products within existing non-smoking policies and improved compliance management. Moreover, reducing density of tobacco retail outlets and limiting tobacco industry influence in the built environment may help prevent indoor tobacco smoking and secondhand smoke exposure in the long term [69].

Finally, while the scope of this analysis and its conclusions were focused on individual-level behavioral sources of indoor PM_2.5_, structural interventions to remove environmental hazards and improve housing quality and stability are ultimately needed to ensure access to health-promoting residential environments more broadly. For example, the upcoming redevelopment of this community will introduce proper kitchen and bathroom ventilation as well as a building shell designed to limit infiltration of particles of outdoor origin.

## Supplementary Material

1

## Figures and Tables

**Table 1:** Questions and response options concerning behavioral variables, from HOME study questionnaires.

Question wording	Response options
Over the past week, how often… …was the stove or oven used for all/any cooking in your home? … did anyone, including yourself and visitors, smoke cigarettes, cigars, or pipes any where inside your home? …were candles used in your home? …was incense used in your home? …were spray air fresheners used in your home? … were any windows in your home open?	Never 1–3 days 4–6 days Every day Don’t know/ Refused
In the past 12 months, how often did you… … use marijuana, which is also called pot or weed?	Never A few times a year Once a month or more Once a week or more Nearly every day Don’t know/Refused

**Table 2:** Demographic characteristics of analytic sample (n = 138).

Characteristic	No. (%)
**Age**	
18–39	35 (26%)
40–64	67 (49%)
65 +	34 (25%)
**Gender**	
Female	108 (78%)
Male	30 (22%)
**Race/Ethnicity**	
Non-Hispanic Black	23 (17%)
Hispanic or Latino	68 (50%)
Non-Hispanic White	31 (23%)
Asian	12 (9%)
Multiracial	2 (1%)
**Language spoken at home**	
English	73 (53%)
Spanish	50 (36%)
Cantonese	9 (7%)
Other/not listed	6 (4%)
**Monthly income $ (mean [SD])**	1913 (1797)
**Education**	
Less than high school	36 (26%)
High school diploma, GED, HiSET, or equivalent	49 (36%)
Some college or Associate’s degree	36 (26%)
4-year or Bachelor’s degree	10 (7%)
Graduate or professional degree	6 (4%)

Note. Percentages were computed using only non-missing responses. For age, race/ethnicity, and education, 1% of sample households had missing values. For monthly income, 12% of sample households had missing values.

**Table 3:** Binary self-reported engagement in PM_2.5_-related behaviors during sampling week, except where noted (n = 138).

Behavior	Yes	No	Missing
**In-unit use, past week** ^a^			
Cooking	111 (80%)	24 (17%)	3 (2%)
Tobacco smoking	25 (18%)	109 (79%)	4 (3%)
Candles	30 (22%)	104 (75%)	4 (3%)
Incense	24 (17%)	110 (80%)	4 (3%)
Air freshener	59 (43%)	73 (53%)	6 (4%)
Window opening	99 (72%)	32 (23%)	7 (5%)
**General use, weekly or more in past year** ^b^			
Cannabis	17 (12%)	116 (84%)	5 (4%)

a Count (percent) of responses coded as yes, no, or missing, based on behavior ever reported in-unit in the past week.

b Count (percent) of responses coded as yes, no, or missing, based on cannabis use reported once a week or more frequently in the past year.

**Table 4:** Results of univariable linear regression models predicting average in-unit PM_2.5_.

Variable	Estimate (95% CI)^a^	p-value
Cooking	1.058 (0.763, 1.466)	0.736
Tobacco smoking	2.563 (1.956, 3.360)	< 0.001*
Candle use	1.363 (1.020, 1.821)	0.038*
Incense use	1.492 (1.098, 2.028)	0.012*
Air freshener use	1.389 (1.088, 1.775)	0.010*
Window opening	1.041 (0.778, 1.393)	0.787
Cannabis use	1.711 (1.185, 2.470)	0.005*
Unit type - townhome	0.738 (0.539, 1.010)	0.060
Ambient PM_2.5_	1.073 (0.963, 1.196)	0.206

* p < 0.05.

a Exponentiated coefficients and 95% confidence intervals represent the multiplicative increase in average in-unit PM_2.5_ associated with a one-unit shift in each predictor.

**Table 5:** Results of multivariable linear regression analysis predicting in-unit average PM_2.5_ from resident behaviors, building type, and outdoor PM_2.5_.

Variable	Estimate (95% CI)^a^	p-value
Cooking	0.891 (0.669, 1.186)	0.429
Tobacco smoking	2.207 (1.633, 2.982)	< 0.001*
Candle use	1.158 (0.888, 1.509)	0.280
Incense use	1.104 (0.818, 1.491)	0.519
Air freshener use	1.219 (0.975, 1.523)	0.084^+^
Window opening	0.832 (0.646, 1.073)	0.159
Cannabis use	1.585 (1.143, 2.199)	0.007*
Unit type – townhome	0.743 (0.566, 0.976)	0.035*
Ambient PM_2.5_	1.047 (0.954, 1.148)	0.336

* p<0.05.

+ p < 0.10

a Exponentiated coefficients and 95% confidence intervals represent the multiplicative increase in average in-unit PM_2.5_ associated with a one-unit shift in each predictor, adjusting for all other model predictors.

**Table 6:** Results of multivariable linear regression with interaction between in-unit past week tobacco smoking and once a week or more cannabis use.

Variable	Estimate (95% CI)^a^	p-value
Cooking	0.888 (0.667, 1.183)	0.419
Tobacco smoking	2.350 (1.692, 3.264)	< 0.001*
Candle use	1.139 (0.872, 1.488)	0.341
Incense use	1.104 (0.817, 1.492)	0.520
Air freshener use	1.218 (0.975, 1.522)	0.085^+^
Window opening	0.842 (0.652, 1.086)	0.187
Cannabis use	1.768 (1.193, 2.62)	0.005*
Unit type – townhome	0.747 (0.569, 0.982)	0.038*
Ambient PM_2.5_	1.047 (0.954, 1.149)	0.335
Tobacco smoking × Cannabis use	0.709 (0.357, 1.41)	0.329

* p < 0.05.

+ p<0.10

a Exponentiated coefficients and 95% confidence intervals represent the multiplicative increase in average in-unit PM_2.5_ associated with a one-unit shift in each predictor, adjusting for all other model predictors. In the case of the interaction between tobacco smoking and cannabis use, the exponentiated coefficient represents the additional multiplicative change in average in-unit PM_2.5_ associated with using both tobacco and cannabis above and beyond the combined independent effects of tobacco and cannabis use, adjusting for all other model predictors.

## Data Availability

The data that support the findings of this study are not publicly available.

## Appendix A. Supporting information

Supplementary data associated with this article can be found in the online version at doi:10.1016/j.indenv.2026.100157.
