# Supplementary material for: In-unit particulate matter (PM2.5) in a public housing complex and the importance of tobacco and cannabis as indoor pollutants
Source: Indoor Environ. Author manuscript; Available in PMC 2026 Apr 16. (PMC13082832; doi:10.1016/j.indenv.2026.100157)
Supplement: 1 [file NIHMS2155816-supplement-1.pdf]

Supplementary material

Text S1. Comparison of PurpleAir $PM_{2.5}$ correction methods.

The geographically closest regional correction method by Koehler et al. (2023) referred to as the Baltimore correction, was developed from PurpleAir data collocated with gravimetric measurements in 151 homes in metropolitan Baltimore, MD. This data was pooled from six studies consisting of adults and children with COPD, HIV, and asthma [67]. Gravimetric measurements were collected via Ultrasonic Personal Air Samplers (UPAS). Individual PurpleAir $PM_{2.5}$ data points with disagreeing channel A and B values were excluded as outlined in Barkjohn et al. (2021) [37] prior to averaging over the sampling week. RH and temperature data were corrected from laboratory-based calibrations prior to application of a second correction (**Eq. (S1)**). The Baltimore correction reduced RMSE by about 40% for mass concentrations <30 $\mu g/m^3$. Koehler et al. also applied the US-wide method to their data set, and although it did not outperform the Baltimore-specific model developed for this study, the US-wide method published by Barkjohn et al. (2021) still substantially improved PurpleAir $PM_{2.5}$ data accuracy [37, 67]. This is a notable finding given that the US-wide correction was developed using outdoor monitoring data, whereas the HOME study measurements presented here were collected in indoor residential settings.

Performance of the US-wide and the Baltimore correction methods were evaluated in a small validation study across 11 households in the target city to match regional conditions of the HOME participants. In each household, a pair of PurpleAir devices were co-located with a pair of gravimetric $PM_{2.5}$ collection devices for two one-week periods. Data were then cleaned and corrected following both the US-wide and the Baltimore correction methods before comparison to the gold-standard gravimetric $PM_{2.5}$ measurements. The US-wide method outperformed both raw and the Baltimore-corrected data, most closely matching gravimetric measurements; hence, we implemented the US-wide method in all analyses.

Eq. (S1). PurpleAir $PM_{2.5}$ correction method developed by Koehler et al., 2023.

$$PM_{2.5,Balt} = a_0 + a_1 \cdot PM_{2.5,u} + a_2 \cdot RH + a_3 \cdot T + a_4 \cdot \textit{weekend} + a_5 \cdot \textit{daytime}$$

Table S1

Sensitivity analyses comparing main model outputs across imputed datasets with 50% (Table 5), 70%, and 90% daily completeness thresholds.

	**50%** (n=138)		**70%** (n=116)		**90%** (n=66)	
**Variable**	**Estimate (95% CI)**[a]	**p-value**	**Estimate (95% CI)**[a]	**p-value**	**Estimate (95% CI)**[a]	**p-value**
Cooking	0.891 (0.669, 1.186)	0.429	0.850 (0.634, 1.140)	0.274	0.910 (0.614, 1.348)	0.632
Tobacco smoking	2.207 (1.633, 2.982)	<0.001*	2.398 (1.740, 3.299)	<0.001*	2.364 (1.515, 3.69)	<0.001*
Candle use	1.158 (0.888, 1.509)	0.280	1.154 (0.869, 1.528)	0.318	0.956 (0.609, 1.502)	0.844
Incense use	1.104 (0.818, 1.491)	0.519	1.005 (0.733, 1.378)	0.973	1.012 (0.604, 1.695)	0.965
Air freshener use	1.219 (0.975, 1.523)	0.084+	1.126 (0.884, 1.433)	0.335	1.201 (0.847, 1.702)	0.297
Window opening	0.832 (0.646, 1.073)	0.159	0.806 (0.609, 1.068)	0.131	0.933 (0.637, 1.367)	0.719
Cannabis	1.585 (1.143, 2.199)	0.007*	1.449 (1.032, 2.034)	0.033*	1.510 (0.952, 2.394)	0.079+
Unit type – townhome	0.743 (0.566, 0.976)	0.035*	0.684 (0.509, 0.919)	0.012*	0.678 (0.445, 1.032)	0.069+
Ambient $PM_{2.5}$	1.047 (0.954, 1.148)	0.336	1.089 (0.983, 1.211)	0.106	1.039 (0.891, 1.212)	0.719

*$p < 0.05$.

[+] $p < 0.10$

[a] Exponentiated coefficients and 95% confidence intervals represent the multiplicative increase in average in-unit $PM_{2.5}$ associated with a one-unit shift in each predictor, adjusting for all other model predictors.

Table S2

Pre-monitoring survey questions and response options for additional sociodemographic, behavioral, and environmental variables used in the imputation model. Questions appear generally in the order administered. All questions also included the response option “Don't know or Refused”, which is not listed here.

**Question wording:**	**Response options**:
What is your date of birth?	• Open response with age at time of survey calculated by study staff
What is your gender?	• Male<br>• Female<br>• Other (specify)
Do you identify as Hispanic, Latino, or Spanish origin? [a]	• Yes<br>• No
What race or races do you identify as? [a]	• White<br>• Black or African American<br>• Asian<br>• American Indian or Alaskan Native<br>• Native Hawaiian or Other Pacific Islander<br>• Multiracial (to be selected only if the respondent states 'multiracial' explicitly)<br>• Other (specify)
What language do you primarily speak at home?	• English<br>• Spanish<br>• Mandarin<br>• Cantonese<br>• Vietnamese<br>• Other (specify)
How many years have you lived in [REDACTED LOCATION]?	• Open response rounded up to nearest integer with less than one year recorded as zero
In the past 12 months, how much of a problem was smelling cigarette smoke coming from other apartments or the hallways?	• Not a problem<br>• Somewhat of a problem<br>• A big problem
What is the highest degree or level of school you have completed?	• Less than 9th grade<br>• 9th to 11th grade

	• High school Diploma, GED, HiSET, or equivalent<br>• Some college, but no degree<br>• 2-Year or Associate's degree<br>• 4-Year or Bachelor's degree<br>• Graduate or professional degree
What was the total income of your household? Include income before taxes from formal and informal employment as well as from other sources such as social security, disability, TANF, or other sources.	• Reported per year or per month, total yearly income calculated by study staff it necessary
Excluding yourself, how many…<br>… adults aged 25 and above live in your household? [b]<br>… adults aged 18 to 24 live in your household? [b]<br>… children under age 18 live in your household? [b]	• 0<br>• 1<br>• 2<br>• 3<br>• 4<br>• 5<br>• 6 or more
How often in the past 12 months…<br>… has anyone including yourself, other household members, or visitors, smoked inside your home?<br>… did you use vapes, e-cigarettes or electronic vape products like JUUL?	• Never<br>• A few times a year<br>• Once a month or more<br>• Once a week or more<br>• Nearly every day
Over the past week how often…<br>… was your home too hot?<br>… was your home too cold?<br>… was air conditioning used in your home?	• Never<br>• 1-3 days<br>• 4-6 days<br>• Every day

[a] Used to calculate composite ethnoracial group variable with the following categories: Asian, Hispanic or Latino, Non-Hispanic Black, Non-Hispanic White, Multiracial, and Other/Not listed. Imputation was performed on the composite value, not separately for each component.
[b] Used to calculate composite household size variable (a total count of all adults and children in the household, including the respondent). Imputation was performed on the composite value, not separately for each component.

Fig. S1. Mean and standard deviation of behavioral variables before dichotomization, by imputation iteration.

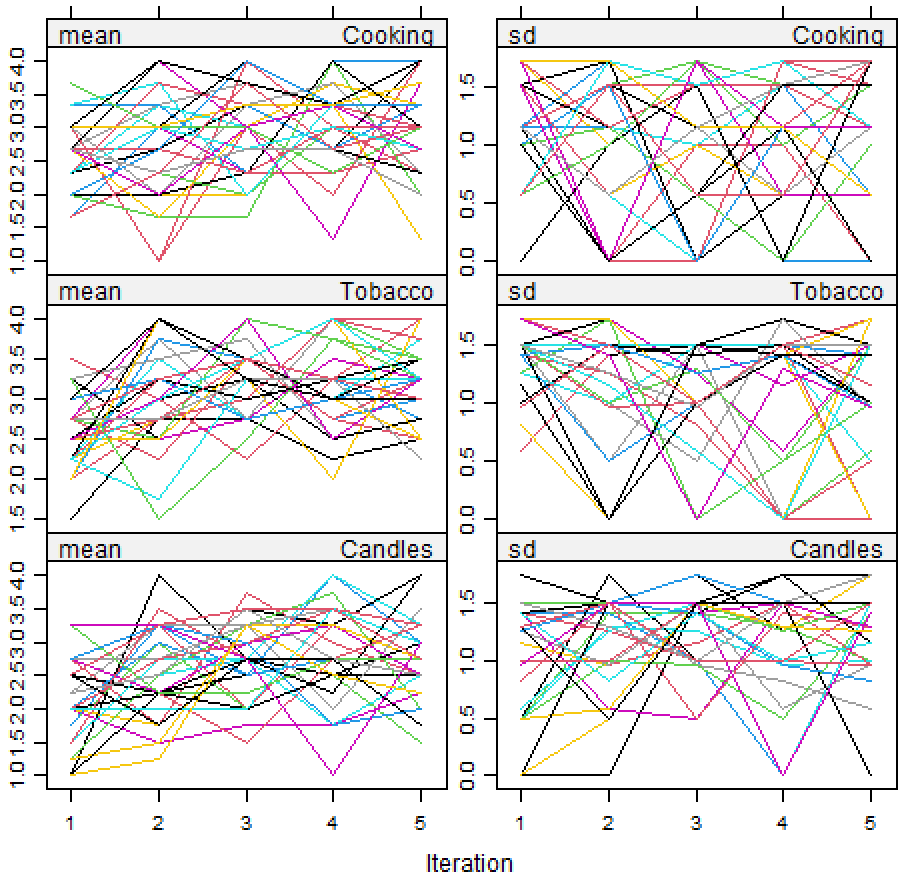

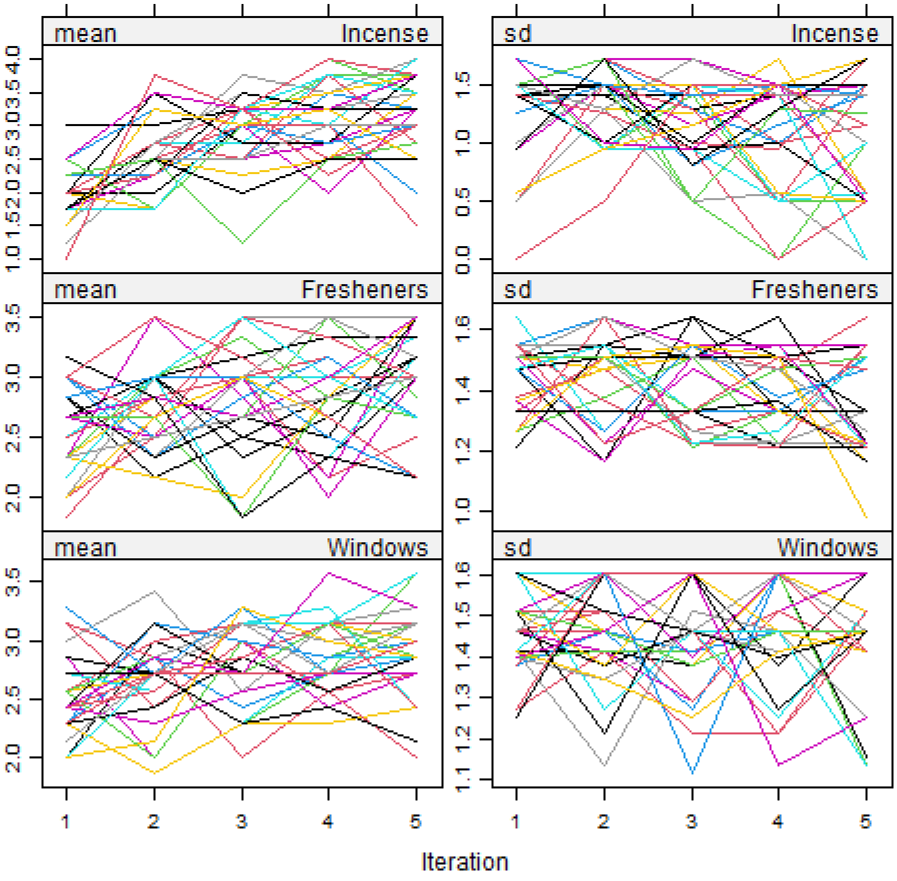
mean
Incense
sd
Incense
mean
Fresheners
sd
Fresheners
mean
Windows
sd
Windows
1
2
3
4
5
Iteration

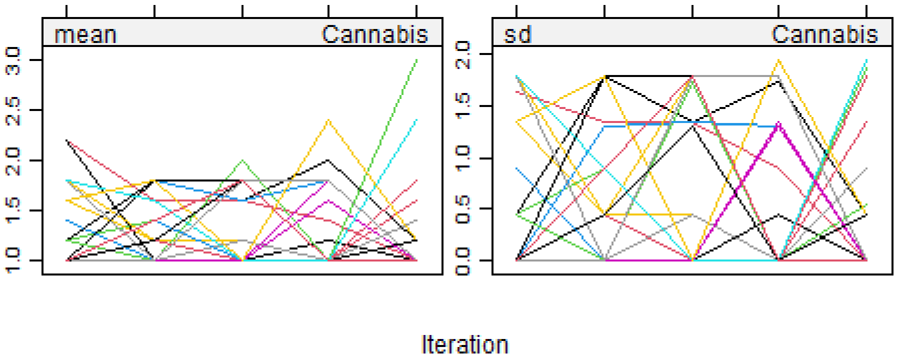
mean
Cannabis
sd
Cannabis
Iteration

Table S3

Imputation diagnostics for variables included in main model.

Variable	RIV	Lambda	FMI
(Intercept)	0.02062	0.02021	0.03572
Cooking	0.04306	0.04128	0.05687
Tobacco smoking	0.0578	0.05464	0.0703
Candle use	0.04052	0.03894	0.05452
Incense use	0.04341	0.0416	0.05719
Air freshener use	0.02108	0.02064	0.03616
Window opening	0.02668	0.02598	0.04151
Cannabis	0.01555	0.01531	0.03082
Unit type – townhome	0.00864	0.00857	0.02407
Ambient $PM_{2.5}$	0.01108	0.01096	0.02646

RIV, Relative Increase in Variance. FMI, Fraction of Missing Information

Table S4

Sensitivity analysis comparing main model outputs across the US-wide corrected data (Table 5), the uncorrected data, and the unimputed data.

	US-Wide corrected (n=138)		Uncorrected (n=138)		Unimputed (n=124)	
**Variable**	**Estimate (95% CI)[a]**	**p-value**	**Estimate (95% CI)[a]**	**p-value**	**Estimate (95% CI)[a]**	**p-value**
Cooking	0.891 (0.669, 1.186)	0.429	0.853 (0.566, 1.288)	0.451	0.958 (0.710, 1.294)	0.777
Tobacco smoking	2.207 (1.633, 2.982)	<0.001*	2.848 (1.822, 4.454)	<0.001*	2.211 (1.627, 2.995)	<0.001*
Candles	1.158 (0.888, 1.509)	0.280	1.221 (0.830, 1.797)	0.313	1.181 (0.909, 1.545)	0.223
Incense	1.104 (0.818, 1.491)	0.519	1.212 (0.787, 1.865)	0.391	1.121 (0.833, 1.510)	0.457
Air freshener	1.219 (0.975, 1.523)	0.084[+]	1.271 (0.915, 1.766)	0.150	1.276 (1.013, 1.600)	0.039*
Window opening	0.832 (0.646, 1.073)	0.159	0.777 (0.559, 1.079)	0.184	0.781 (0.598, 1.018)	0.067[+]
Cannabis use	1.585 (1.143, 2.199)	0.007*	1.822 (1.132, 2.933)	0.015*	1.586 (1.142, 2.199)	0.007*
Unit type – townhome	0.743 (0.566, 0.976)	0.035*	0.627 (0.422, 0.932)	0.023*	0.710 (0.537, 0.945)	0.020*
Ambient $PM_{2.5}$	1.047 (0.954, 1.148)	0.336	1.062 (0.920, 1.225)	0.387	1.056 (0.959, 1.153)	0.267

*$p < 0.05$.

[+] $p < 0.10$

[a] Exponentiated coefficients and 95% confidence intervals represent the multiplicative increase in average in-unit $PM_{2.5}$ associated with a one-unit shift in each predictor, adjusting for all other model predictors.

Table S5
Pearson correlation coefficients among multivariable model predictors, including binary behavioral variables, binary building type, and ambient average $PM_{2.5}$ concentration.

	Cooking	Window opening	Tobacco smoking	Candles	Incense	Air fresheners	Cannabis	Unit type – townhome	Ambient $PM_{2.5}$
Cooking	—	0.087	0.173	-0.096	0.058	0.028	0.117	-0.116	-0.025
Window opening		—	0.104	0.152	0.047	0.159	0.068	-0.156	0.175
Tobacco smoking			—	0.092	0.313	0.222	0.150	-0.085	0.130
Candles				—	0.248	0.083	-0.006	-0.076	0.069
Incense					—	0.040	-0.077	0.029	0.031
Air fresheners						—	-0.132	-0.065	0.103
Cannabis							—	-0.070	0.020
Unit type – townhome								—	-0.011
Ambient $PM_{2.5}$									—
